# The case of the missing spacer!

**DOI:** 10.1093/jscr/rjad163

**Published:** 2023-03-28

**Authors:** Andrea Boerkamp, Marie Shella De Robles

**Affiliations:** Department of Surgery, Shoalhaven District Memorial Hospital, Nowra, New South Wales 2541, Australia; Graduate School of Medicine, University of Wollongong, Keiraville, 2500 New South Wales, Australia; Department of Surgery, Shoalhaven District Memorial Hospital, Nowra, New South Wales 2541, Australia; Department of Surgery, Wollongong Hospital, Wollongong, 2500 New South Wales, Australia; Graduate School of Medicine, University of Wollongong, Keiraville, 2500 New South Wales, Australia

## Abstract

The SpaceOAR Vue hydrogel system was developed to reduce the toxicity to the rectum following radiation therapy for prostate cancer. Initial trial data reported the product as overall effective and safe. However, a few additional observed complications have likely been brought on by its increased utilization. The case presented herein describes rectal erosion, with abscess and rectal fistula formation, associated with the use of the SpaceOAR Vue hydrogel system. The SpaceOAR Vue hydrogel system was subsequently found to be absent between radiotherapy treatments and was thought to have been passed rectally through the fistula. The benefits and complications of the SpaceOAR Vue hydrogel system are discussed, as well as key factors to consider as the recommendation of routine use increases.

## INTRODUCTION

The SpaceOAR Vue hydrogel system is designed to provide protection of the rectum during radiotherapy of the prostate gland. SpaceOAR Vue is a radio-opaque version of the SpaceOAR hydrogel system. The solution is injected through the perineum into the space between the prostate and rectum, thereby increasing the distance between the two structures [1]. The SpaceOAR Vue system has shown benefits such as a significant reduction in the rectal radiation dose and lower rates of rectal toxicity in patients undergoing radiotherapy for prostate cancer [2]. Safety performance in initial trials was promising, with few adverse outcomes. However, SpaceOAR Vue remains in early clinical use [3, 4]. Given the early stages of its use and the case discussed herein, we examined emerging safety reports as the system becomes more widely utilized.

## CASE REPORT

A 72-year-old male with a family history of prostate cancer presented with an elevated prostate-specific antigen (PSA) and was found to have an asymmetrically enlarged firm prostate on digital rectal examination. Subsequent magnetic resonance imaging (MRI) showed Prostate Imaging Reporting & Data System 4 lesions. Biopsies confirmed adenocarcinoma in 2 of 38 cores, with a Gleason score of 3 + 4 = 7. He was planned for radiotherapy.

The patient underwent placement of fiducials and SpaceOAR Vue prior to treatment. He also had a colonoscopy to investigate a recent positive occult blood test 1 week after the insertion of the spacer. A single caecal polyp was removed, and the rectum was noted to be normal during the colonoscopy. Both procedures were straightforward and without immediate complications.

**Figure 1:**
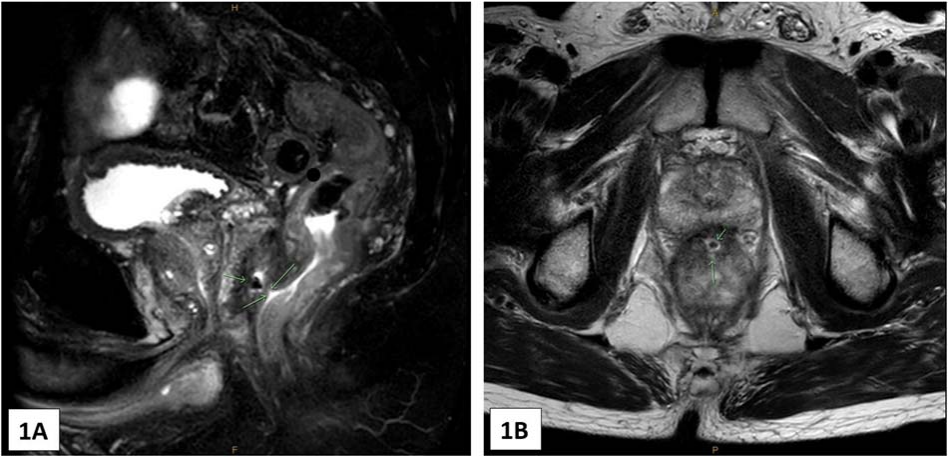
(**A**) and (**B**)—MRI images (A in sagittal, B in axial) showing the 3 mm tract from the low rectum extending into the recto-prostatic angle. This is in connection with a small collection at the recto-prostatic angle.

Radiotherapy was commenced 3 weeks following the spacer insertion, with a dose plan of 60 Gy divided into 20 fractions. Prior to commencing the 12th fraction, it was noted that the SpaceOAR Vue had disappeared. The patient had also noticed passing a gel-like substance per rectum in the days preceding this discovery. There had also been a single episode of small-volume rectal bleeding. Radiotherapy was ceased, and an MRI pelvis was completed, which showed a 3 mm sinus between the recto-prostatic angle and the low rectum (Fig. 1A and B). In addition, there was a small collection at the recto-prostatic angle, which was accompanied by significant granulation and fibrous tissue.

**Figure 2:**
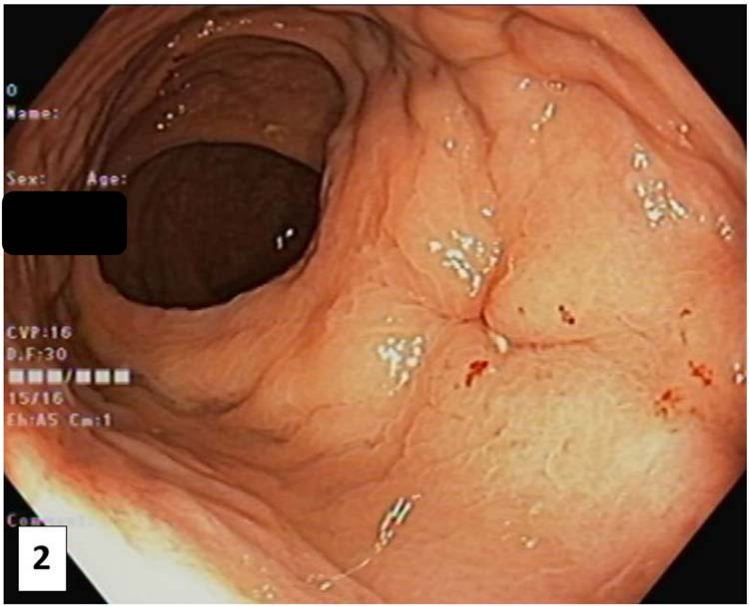
Photograph of the anterior rectal wall taken at colonoscopy after the SpaceOAR Vue was noted to be missing. This lesion, likely a healing sinus, corresponds to the location of the abnormality of the pelvic MRI.

The patient was referred to a colorectal surgeon who performed a repeat colonoscopy and examination of the anorectum under anaesthesia. These showed a healing sinus at the anterior low rectum (Fig. 2), coinciding with the region of interest described in the MRI. At this point, the patient was asymptomatic.

The patient received 36 Gy of the planned 60 Gy in 12 fractions. Radiotherapy was abandoned given the complication of rectal wall erosion. Fortunately for this patient, this complication was able to be managed conservatively. Following discussions regarding alternative treatments for his prostate cancer, such as prostatectomy and androgen deprivation therapy, the patient opted to monitor the PSA trajectory for the foreseeable future and avoid further active management.

## DISCUSSION

Prostate cancer is a common malignancy, with radiotherapy being one of the main curative or palliative treatments offered. The SpaceOAR Vue hydrogel system was licenced for use by the Food and Drug Administration (FDA) in 2015 and was developed in response to the incidence of complications arising from immediate and delayed rectal radiation toxicity [5]. Rectal and genitourinary toxicity represents a significant disease burden following pelvic radiation and is a costly complication for health services [6]. The cost-effectiveness of hydrogel spacing systems (including SpaceOAR Vue) was evaluated, showing a marginal increase in cost when used in conformal radiotherapy treatment but coupled with a significant reduction in rectal toxicity. When used in high-dose radiotherapy, it was a cost-effective measure [7]. These results have favoured increasing the use of rectal spacers such as SpaceOAR Vue. However, costs associated with the increasing incidence of severe complications are yet to be considered.

A paper published by Aminsharifi *et al*. in 2019 [3] compared complications listed on the SpaceOAR Vue manufacturer’s website to those reported in the literature and on the Manufacturer and User Facility Device Experience (MAUDE) database. The MAUDE database is an FDA-maintained database where device complications can be anonymously reported voluntarily. This study found a discrepancy between the severity of complications reported by the manufacturer and those reported by clinicians in the field [3].

Manufacturer reported complications include pain, inflammation, infection, urinary retention, constipation, rectal/bladder/prostate perforation, fistula, mucosal damage/ulcers, necrosis, allergy and embolism [1]. Reports of increasingly serious complications have been published since the product received its licencing [2, 3]. Although the incidence of more serious complications remains low, the severity of such complications needs to be considered by clinicians prior to SpaceOAR Vue being recommended as a routine part of treatment.

Evidence suggesting the proposed mechanism for the formation of rectal fistulae is lacking. If this complication occurred early following SpaceOAR Vue insertion, as reported here, it could be associated with a difficult or traumatic insertion. Difficulties were not reported as a factor in the case discussed. Inflammation and tissue reaction in response to the hydrogel may be a factor and could explain why some patients develop certain complications whilst others do not. Similarly, it is possible that the radiation itself caused the formation of the rectal fistula. However, this is incredibly rare this early in treatment [8]. There may be contributing factors from each of the above scenarios leading to fistula formation.

Important clinical benefits are reported to result from the use of the SpaceOAR Vue system [9, 10]. Despite this, cases of severe complications exist arising from the use of the product [2, 3]. Prostate cancer is a slow-growing malignancy, meaning many patients will succumb to other illnesses, and prostate cancer will not cause death. In light of this, any treatments offered for prostate cancer should provide a robust and measurable benefit prior to being offered as a routine therapy. The increasing clinical use of the SpaceOAR Vue system should provoke further investigation of mechanisms for severe complications associated with its use, thereby allowing clinicians to identify patients who may be at an increased risk of severe complications.

## CONFLICT OF INTEREST STATEMENT

None declared.

## FUNDING

None.
